# Investigating the Impact of *Abelmoschus esculentus* on Glycemia and Insulin Resistance in Type 2 Diabetes and Prediabetes

**DOI:** 10.3390/plants15050817

**Published:** 2026-03-07

**Authors:** Kabelo Mokgalaboni, Wendy N. Phoswa, Sidney Hanser, Sogolo L. Lebelo

**Affiliations:** 1 Department of Life and Consumer Sciences, College of Agriculture and Environmental Sciences, University of South Africa, Florida Campus, Florida 1710, South Africa; phoswwn@unisa.ac.za (W.N.P.); lebelol@unisa.ac.za (S.L.L.); 2 Department of Physiology, School of Medicine, Sefako Makgatho Health Science University, Ga-Rankuwa 0208, South Africa; sidney.hanser@smu.ac.za

**Keywords:** *Abelmoschus esculentus*, glycemic control, insulin resistance, okra, prediabetes, type 2 diabetes

## Abstract

**Background:** *Abelmoschus esculentus* L. (okra) has shown potential efficacy in animal models of metabolic disorders; however, evidence from clinical studies emanates from trials with a small sample size, and the findings remain contradictory. This study aims to evaluate the impact of okra on glycemia and insulin resistance in individuals with type 2 diabetes (T2D) and prediabetes. **Method:** A literature search was conducted on PubMed, Scopus, ScienceDirect, and Web of Science, including manual screening of references. *Abelmoschus esculentus*, okra, *Hibiscus esculentus*, lady’s finger, and diabetes were used as potential keywords and adjusted for each database. A meta-analysis web tool was used to analyze the data, with results reported as the mean difference (MD) or standardized mean difference (SMD), along with 95% confidence intervals (CI). **Results:** Nineteen clinical studies conducted in patients with T2D and prediabetes were analyzed. The evidence revealed that, compared to the control group, okra significantly reduced fasting blood glucose (SMD = −0.70 (95% CI, −1.03 to −0.36), *p* < 0.0001) and glycated hemoglobin (MD = −0.77%, 95%CI (−1.36 to −0.18), *p* = 0.0102. Furthermore, it reduced the homeostatic model assessment of insulin resistance (HOMA-IR) levels, MD = −0.61, 95% CI (−1.07, −0.15), *p* < 0.0097. However, no significant effect was observed on insulin (*p* = 0.5823). **Conclusions:** The evidence gathered in this study suggests that okra may have the potential to regulate glycemia in individuals with T2D and prediabetes. However, the effect on insulin resistance remains controversial, as only HOMA-IR was improved.

## 1. Introduction

Non-communicable diseases remain the leading cause of mortality globally [1]. These include cardiovascular diseases, diabetes mellitus, and are associated with a high risk of stroke, heart attack, heart failure, and peripheral artery disease. According to the International Diabetes Federation (IDF), the global prevalence of diabetes was 588.7 million in 2024, with an estimated increase to 852.5 million by 2050 [2]. While in Africa, the prevalence was low (24 million) in 2024, it is estimated to reach 59.5 million by 2050, representing a 145% increase compared to other continents.

**Figure 1:**
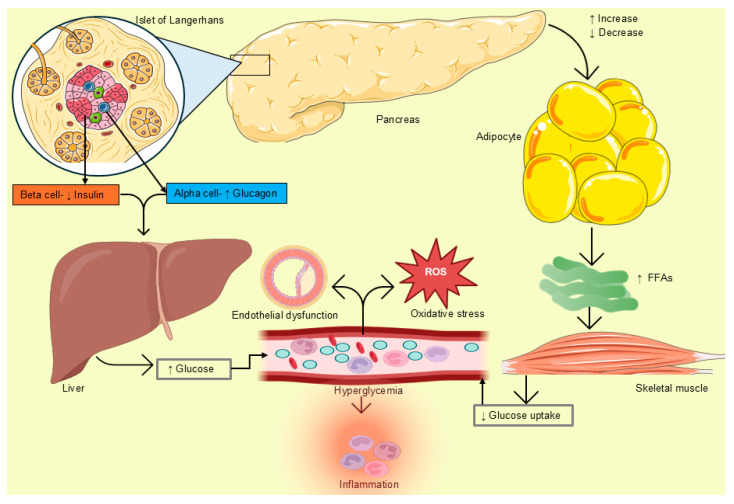
Pathogenesis of hyperglycemia in diabetes mellitus [4]. FFAs: Free fatty acid. Created using Bioicons, BioRender, and Microsoft PowerPoint.

Hyperglycemia is a central feature of diabetes mellitus. This arises from impaired pancreatic islet function, which subsequently promotes hepatic glucose production, thereby increasing blood glucose levels [3,4]. This can also be attributed to increased free fatty acid production from adipose tissue, which inhibits glucose uptake in skeletal muscle, thereby promoting hyperglycemia (Figure 1). In type 2 diabetes (T2D), its persistence is associated with secondary complications, including oxidative stress and nephropathy [5]. Given that the global prevalence of T2D is rising annually, the financial burden on the healthcare system could be even greater, especially in middle- and low-income countries. For instance, a 2017 health executive commission stated that approximately 60% of individuals from low- and middle-income countries cannot afford cardiovascular medicines [6]. For instance, the cost of T2D treatment in healthcare facilities in South Africa was approximately 2.7 billion rand in 2018 [7]. In Kenya, the estimated amount was 74,521 million Kenyan shillings in 2021 [8]. Indeed, these costs are too high, making it difficult for this group of patients to receive adequate medical attention, especially in Africa. While this is the case, it is essential to recognize the use of plant-based remedies in Africa, as also supported by the World Health Organization [9]. Africa is rich in some of these plants, which have been used for a long time to treat various conditions, including diabetes [10,11,12]. Although in some cases they are not scientifically proven, users reported evidence supporting their symptom-relieving effects.

One of these important plants is okra, also known as ladies’ fingers, and is scientifically classified as *Abelmoschus esculentus* L. or *Hibiscus esculentus* [13]. This plant is a member of the Malvaceae family and is rich in active phytochemicals, including alkaloids, anthraquinones, cardenolides, flavonoids, glycosides, phenolics, saponins, steroids, tannins, and terpenoids, which may contribute to its antidiabetic properties [14,15]. A systematic review of preclinical studies has shown that the okra fruit extract or its juice has a positive effect in controlling hyperglycemia by reducing glycated hemoglobin (HbA1c), homeostatic model assessment of insulin resistance (HOMA-IR), oral glucose tolerance test, and fasting blood glucose (FBG) [16]. However, while these findings may be relevant to diabetes, it is also important to note the limitations in translating preclinical findings to clinical settings. Our research team previously reported the effect of okra on FBG in prediabetics and T2D; however, no effect on HbA1c was observed, rendering the conclusion on hyperglycemia less valid, as other parameters were not assessed [17]. Although other meta-analyses have shown potential benefits in various markers of hyperglycemia, it is essential to acknowledge their limitations. For example, Jafari et al. (2025) demonstrated an effect on FBG, HbA1c, insulin, and HOMA-IR among individuals regardless of their health status [18]. However, another study reported contradictory findings, showing an effect of okra on FBG and HbA1c, and a null effect on HOMA-IR and insulin, among individuals with diabetes and prediabetes [19]. The latter findings are supported by a more recent study of the same design, which showed that okra reduces FBG and HbA1c, but not insulin or HOMA-IR [20]. This study was based on six trials, which may have limited its statistical power. Overall, other studies suggest that okra may be beneficial in glycemic control; the contradictory findings imply a potential limitation of okra in fully addressing hyperglycemia. It is also important to note that they did not simultaneously measure all markers of glycemic control, making it challenging to draw conclusions about the glycemic status after okra supplementation. Therefore, the current study aims to evaluate the effects of okra on glycemic markers and insulin resistance in individuals with T2D and prediabetes.

## 2. Methodology

This is a quantitative study that uses secondary data from the published literature and was registered with PROSPERO (CRD420251180683). It adhered to PICO criteria as previously reported [21]. The study was considered relevant if it included patients receiving any form of okra treatment; the outcomes were markers of glycemia and insulin resistance (FBG, HbA1c, insulin, and HOMA-IR). It is reported following the Preferred Reporting Items for Systematic Review and Meta-Analysis (PRISMA) [22] (Supplementary File S1).

### 2.1. Literature Search and Sources

The search for relevant studies was conducted by two independent researchers (K.M. and W.N.P.) on PubMed, Scopus, ScienceDirect, and Web of Science (Supplementary File S2 Table S1). The keywords used in the search builder included ‘okra’ alone or together with associated synonyms such as ‘lady’s finger’, ‘*Abelmoschus esculentus*’, ‘*Hibiscus esculentus*’, and the condition ‘diabetes’. The Boolean operators, “OR” and “AND”, were used to combine synonyms and interventions with conditions, respectively. No restriction in terms of study design or language, except for the year of publication, as the search was filtered for publications from inception until 22 October 2025. The online Google Translate tool was used to translate studies published in languages other than English, thereby minimizing the language-associated risk of bias [23].

### 2.2. Inclusion and Exclusion Criteria

The eligibility of the included studies was determined by whether they met the preplanned PICO criteria. Hence, those included were conducted among individuals with prediabetes and T2D, used okra as an intervention, and measured at least one of the markers of hyperglycemia. On the other hand, those conducted in other conditions, including healthy individuals and rodent models of diabetes, were all excluded from this study. To minimize the risk of selection bias, conference proceedings with sufficient data were also considered for inclusion in alignment with previous recommendations [24].

### 2.3. Quality Assessment

The Cochrane risk of bias tool (ROB) and risk of bias in non-randomized studies of intervention [25,26] were used to assess all domains by independent researchers (KM and WNP). The overall quality was classified as good, moderate, or poor based on the scores of each domain.

### 2.4. Data Items and Extraction

From each relevant study, KM and WNP independently extracted information such as the author’s last name and year of publication, the condition investigated, sample size in each group, demographic information (including gender, age, and BMI), and intervention details (type of okra used, dosage, and duration of administration). In addition, we extracted data for each outcome measure (FBG, HbA1c, insulin, and HOMA-IR).

### 2.5. Data Synthesis and Analysis

For a meta-analysis to be conducted for each outcome, one condition had to be met: the presence of at least two studies. Otherwise, narrative reporting was adopted. Data from each study regarding the outcome variables were collected as either the mean and standard deviation (SD), the mean and interquartile range (IQR), or the median and range. However, the final data were transformed into mean and SD for analysis. Therefore, the data reported as median and range were converted to mean and SD using the online calculator described by Hozo et al., 2005 [27]. In studies that reported means and IQRs, SD was estimated using the formula SD = IQR/1.35. To determine the difference between the intervention and control groups, we estimated the mean change by subtracting each group’s final mean from its baseline. We also estimated the change in SD by using the formula from the Cochrane Handbook, change in SD = square root of (SD_b_)^2^ + (SD_f_)^2^ − 2 * R*SD_b_*SD_f_ [28]. The subscripts b and f denote baseline and final, respectively, while R denotes the correlation coefficient, set to 0.5 throughout these calculations, consistent with previous studies. The meta-analysis was conducted using the online meta-analysis web tool [29]. The data were reported as the mean difference (MD) or standardized mean difference (SMD) with 95% confidence intervals (CI), depending on whether the same or different units of measure were used, respectively. The effect size was interpreted using Cohen’s d, with d values of 0.1, 0.5, and 0.8 considered small, medium, and large, respectively [30]. For evidence with low heterogeneity (*I*^2^ < 15%), the fixed-effect model was used, assuming less variation across studies. Otherwise, the random-effect model was used if high heterogeneity (*I*^2^ > 50%) was observed, followed by subgroup analysis to investigate the source of this variation. Publication bias was assessed through a visual inspection of the funnel plot, with funnel symmetry indicating the absence of bias. The Eggers test (*p* > 0.05) also suggested the absence of bias. Subgroup analyses were conducted based on the study’s quality and the country in which the study was conducted. Sensitivity analysis was conducted to confirm the stability of the observed effect size using a one-study exclusion method [31].

## 3. Results

### 3.1. Literature Sources and Screening Procedure

**Figure 2:**
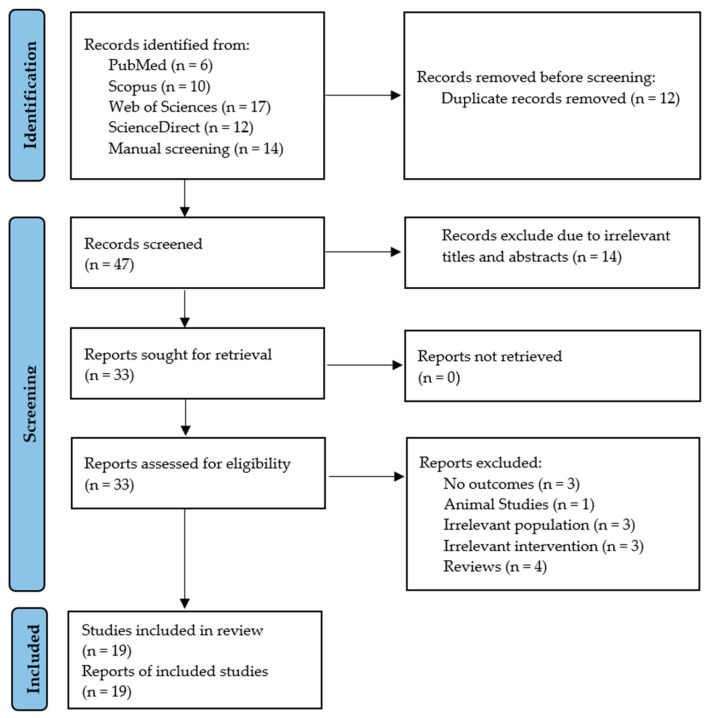
PRISMA flow diagram.

From the four online databases and search engines, at least 45 records were identified, and an additional fourteen records were identified through non-standardized methods, such as manual screening of relevant records. A screening procedure was conducted in accordance with the PRISMA flow diagram guideline, as outlined in Figure 2. Of the identified records, twelve were deemed duplicates after grouping them in ascending order in an Excel sheet. Of the 47 that underwent initial screening of the title, abstract, and keywords, 14 were excluded as irrelevant. The second phase of screening, which involved a full-text evaluation, identified fourteen records as irrelevant because they did not meet the preplanned PICO criteria. The noted reasons included studies that did not report the outcome of interest, were not conducted in individuals living with prediabetes or T2D, did not use okra as an intervention, were studies in rodent models, or were full-text articles presenting findings from a review study. Therefore, only 19 studies [32,33,34,35,36,37,38,39,40,41,42,43,44,45,46,47,48,49,50] met the PICO criteria and were thus included in the final analysis.

### 3.2. Characteristics of Included Studies

A total of 19 [32,33,34,35,36,37,38,39,40,41,42,43,44,45,46,47,48,49,50] clinical studies published in peer-reviewed journals between 2011 and 2025 were considered relevant and included in the meta-analysis. Fifteen studies were published in the previous 6 years, with 26% published in 2025. Eighteen studies were published in patients with T2D, with two studies having T2D concomitant with DN [39] or hypercholesterolemia [44]. Only one study was conducted among patients with prediabetes [32]. Six studies employed a quasi-experimental design with pre- and post-treatment follow-ups. Of the 13 studies, 5 used a double-blinded randomized controlled trial, two used a triple-blinded randomized controlled trial, and one used a single-blinded randomized controlled trial. In contrast, others did not report whether the study was single or double-blind. In terms of distribution, seven were conducted in Iran, five in India, four in Indonesia, and one in each of the following countries: Pakistan, China, and Bangladesh. The administration of okra varied across studies, with some using okra fruit juice, soaked okra water, okra powder in a meal, or powdered capsules. The sample size in each study ranged from the smallest (10) [49] to the largest (200) [47]. The age, gender, and BMI of included participants are presented in Table 1.

### 3.3. Effect of Okra on Hyperglycemic Parameters

#### 3.3.1. Effect on Glycemic Control

**Figure 3:**
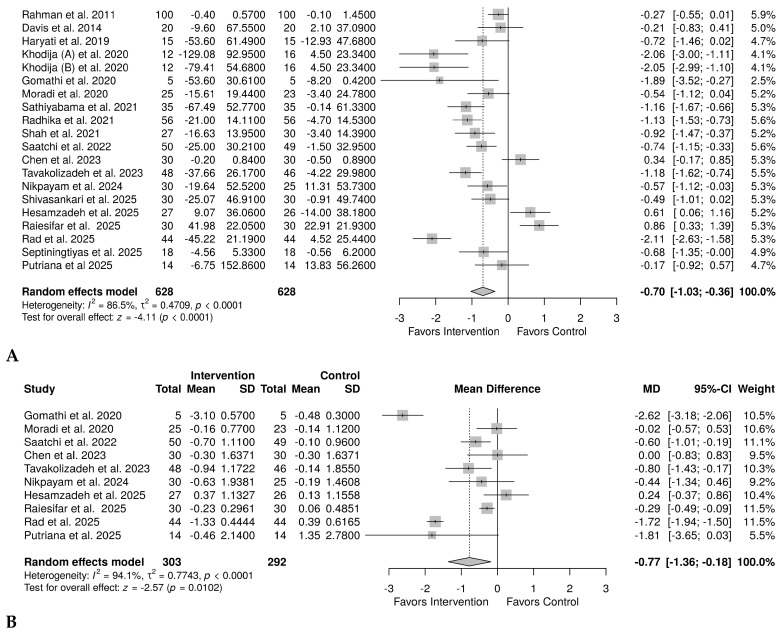
Random effect model meta-analysis forest plot showing the effect of okra on glycemic control in prediabetes and T2D. (**A**). Change in the levels of fasting blood glucose in 614 individuals on okra and 614 in the control group, SMD= −0.73, 95% CI (−1.10 to −0.36), *p* = 0.0001 [32,33,34,35,36,37,38,39,40,41,42,43,44,45,46,47,48,49,50]. (**B**). Change in glycated hemoglobin in 289 individuals in the okra group compared to the 278 in the control group, MD = −0.71%, 95% CI (−1.32 to −0.10), *p* = 0.0220 [32,35,36,37,39,40,41,42,49,50].

A total of 19 studies [32,33,34,35,36,37,38,39,40,41,42,43,44,45,46,47,48,49,50] provided sufficient information about the effect of okra on FBG in both groups. One study [44] had used okra in two forms (steamed and boiled) and was thus treated as two sub-studies. Due to variation, as quantified by *I*^2^ = 86.5%, a random-effects model meta-analysis was adopted, and the results showed a significant effect of okra in reducing FBG, with an effect size SMD = −0.70 (95% CI, −1.03 to −0.36), *p* < 0.0001 (Figure 3A). Ten studies [32,35,36,37,39,40,41,42,49,50] were analyzed for HbA1c; similarly, a random-effects model meta-analysis was used due to high heterogeneity (*I*^2^ = 94.1%). The results showed a medium yet significant effect of okra in reducing HbA1c, MD = −0.77%, 95% CI (−1.36 to −0.18), *p* = 0.0102 (Figure 3B).

#### 3.3.2. Effect on Insulin Resistance

**Figure 4:**
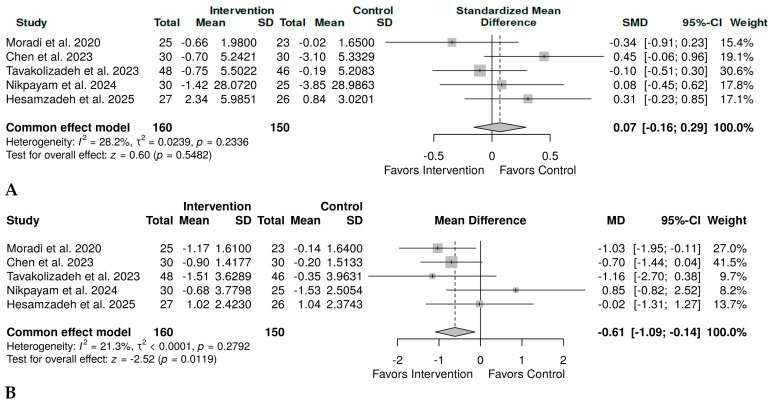
Fixed-effect model meta-analysis forest plot showing the effect of okra on insulin resistance in prediabetes and T2D. (**A**). Change in the levels of insulin in 160 individuals in the okra group and 150 in the control group, SMD = 0.07, 95% CI (−0.16 to 0.29), *p* = 0.5482 [32,37,39,40,42]. (**B**). Change in homeostatic model assessment of insulin resistance in 160 individuals in the okra group compared to 150 individuals in the control group, MD = −0.61, 95% CI (−1.09 to −0.14), *p* = 0.0119 [32,37,39,40,42].

Five studies [32,37,39,40,42] were analyzed to compare the effect of okra on insulin levels with that of the control group. The overall results revealed a small, non-significant effect of okra on insulin, SMD = 0.07, 95% CI (−0.16 to 0.29), *p* = 0.5482 (Figure 4A). However, there was minimal heterogeneity (*I*^2^ = 28.2%); therefore, fixed-effect meta-analyses were conducted. The same number of studies [32,37,39,40,42] was analyzed for HOMA-IR. Due to lower heterogeneity (*I*^2^ = 21.3%), fixed effect model meta-analysis was performed, and the results showed a medium to large effect size, MD = −0.61, 95% CI (−1.09 to −0.14), *p* = 0.0119 (Figure 4B).

### 3.4. Publication Bias Assessment

**Figure 5:**
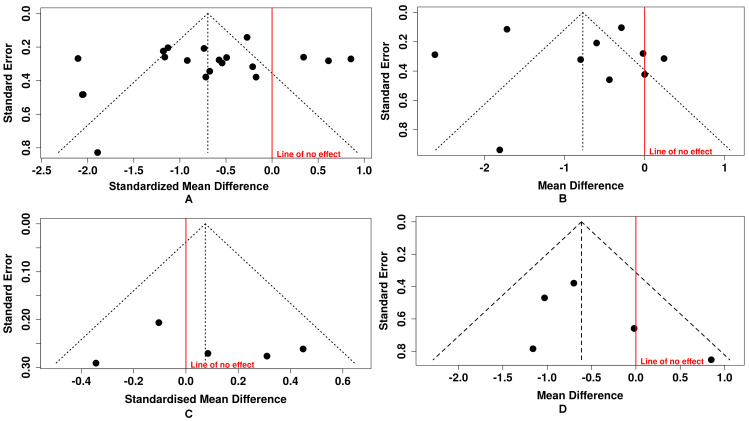
Symmetrical funnel plots in the assessment of publication bias. (**A**) Funnel plot evaluating publication on fasting blood glucose, 19 studies with 20 different treatment arms and 1256 participants (Egger test *p* = 0.30). (**B**) Funnel plot evaluating publication on glycated hemoglobin; ten studies with 595 participants (*p* = 0.722). (**C**) Funnel plot evaluating publication on insulin, five studies with a sample of 310 participants (Egger’s test, *p* = 0.759); (**D**) Funnel plot evaluating publication on homeostatic model assessment of insulin resistance, with five studies and 310 participants (Egger’s test, *p* = 0.363). The dots in each funnel plot represent individual studies, the central dotted lines indicate the overall pooled effect, and the diagonal dotted lines show the 95% confidence intervals.

The assessment of publication bias across the included outcomes showed a symmetrical distribution in the funnel plots, suggesting no bias. Specifically, for FBG, the funnel plot showed a symmetrical shape (Figure 5A), and this was supported by Egger’s test, which yielded an intercept of −2.13 (95% CI: −6.04 to 1.78), *p* = 0.30. Similarly, for HbA1c, both the visual inspection of funnel plot (Figure 5B) and Egger’s test (intercept: 1.16, 95% CI: −4.96 to 7.28, *p* = 0.722) indicated no evidence of publication bias. The analysis of insulin revealed the same trend (Figure 5C), with Egger’s test showing an intercept of 1.58 (95% CI: −7.65 to 10.81), *p* = 0.759, further supporting the absence of publication bias. Likewise, HOMA-IR demonstrated no notable asymmetry in the funnel plot (Figure 5D) and Egger’s test (intercept = 1.74, 95% CI (−1.45 to 4.94), *p* = 0.363 confirmed these findings. Altogether, these findings suggest that the meta-analysis results are unlikely to have been influenced by selective reporting or small-study effects, supporting the credibility of the pooled estimates.

### 3.5. Sensitivity Analysis

The overall results of the sensitivity analysis are presented in the Supplementary File S2 Table S2. The sensitivity analysis showed that the pooled effect size remained stable, ranging from −0.64 to −0.78 when individual studies were excluded sequentially, compared with the initial effect size of −0.71 for FBG. The direction and statistical significance of the effect remained unchanged, suggesting that no single study influenced the overall results. On the other hand, the exclusion of a study by Gomathi et al. [49], which used both okra and metformin, led to a change in the effect size (MD = −0.55), a 28.6% decrease from the initial effect size of −0.77. Despite this fluctuation, the direction and statistical significance of the effect size remain consistent, suggesting that the overall results were robust. Moreover, for insulin, sensitivity analysis showed that the pooled effect fluctuated between −0.02 and 0.14 after sequential exclusion of individual studies, compared with the original effect size of 0.07. For HOM-IR, excluding individual studies did not change the effect size, direction, or statistical significance, suggesting the results were more robust.

### 3.6. Subgroup Analysis

For FBG, based on country of publication and study quality, we found that studies published in India and moderate-quality RCTs contributed to the observed heterogeneity (Supplementary File S2 Table S3). Participants’ condition did not affect heterogeneity. For HbA1c, condition, study quality, and country of publication, none influenced heterogeneity (Supplementary File S2, Table S3).

### 3.7. Risk of Bias and Quality of Studies

The risk of bias of twelve RCTs is presented in Supplementary File S2 Figure S1A. In brief, four studies did not clearly describe the randomization method and were therefore classified as high risk in this domain. Seven of the trials sufficiently reported the randomization process and were thus classified as low risk of bias in the first domain. Of these studies, three were classified as having some concerns for the fifth domain, as no protocol or trial registration was found online or stated in the article. For seven non-randomized studies (Supplementary File S2 Figure S1B), five studies with (6 arms) were regarded as having low quality due to a serious risk of bias across different domains. Only two studies were considered to have moderate quality.

## 4. Discussion

The current study found that okra supplementation in patients with prediabetes and T2D significantly improved FBG, HbA1c, and HOMA-IR. However, no effect was observed on insulin levels. These patients are characterized by hyperglycemia and insulin resistance, factors that promote inflammation, oxidative stress, the development of secondary complications, and CVD-related mortality. As okra has been reported to reduce glycemia and insulin resistance, it can be considered an adjunctive therapy to control hyperglycemia in this group of patients.

It is worth noting that the effect of okra on FBG in prediabetics and T2D is consistent with our previous findings [17]. However, the earlier study showed no effect on HbA1c and did not assess effects on insulin or HOMA-IR, which remain central markers of hyperglycemia. Among other confounding factors that might have contributed to the null effect on HbA1c is the inclusion of small clinical studies, which reduced the statistical power. The current study builds on previous evidence and confirms okra’s potential to reduce various markers of hyperglycemia, focusing on different markers and improving statistical power by including 19 studies. A more recent quantitative study also suggests that okra may be a potential agent in ameliorating FBG and HbA1c [20]. However, it is worth noting that this study found no effect on HOMA-IR or insulin levels. Suggesting a potential limitation attributable to the sample size and the population included.

In metabolic disorders, elevated HOMA-IR is associated with insulin resistance and hyperglycemia, which contribute to complications in diabetes [51]. Interestingly, in the current analysis, okra alleviated insulin resistance, as demonstrated by a reduction in HOMA-IR, suggesting improved insulin sensitivity and glucose handling [32,40,42]. The result is supported by findings from Nikpayam et al. (2024), who demonstrated a decrease of at least 17.26% in HOMA-IR in the diabetes group after okra treatment compared to baseline [39]. This effect may be attributed to the combined use of okra and both magnesium stearate and Avicel. Normally, Avicel enhances drug absorption, while magnesium is also recognized for its antihyperglycemic properties [52,53]. The overall results suggest that the β-cells of the pancreas’s islets of Langerhans are no longer overproducing insulin. This preserves pancreatic function and ameliorates insulin resistance.

**Figure 6:**
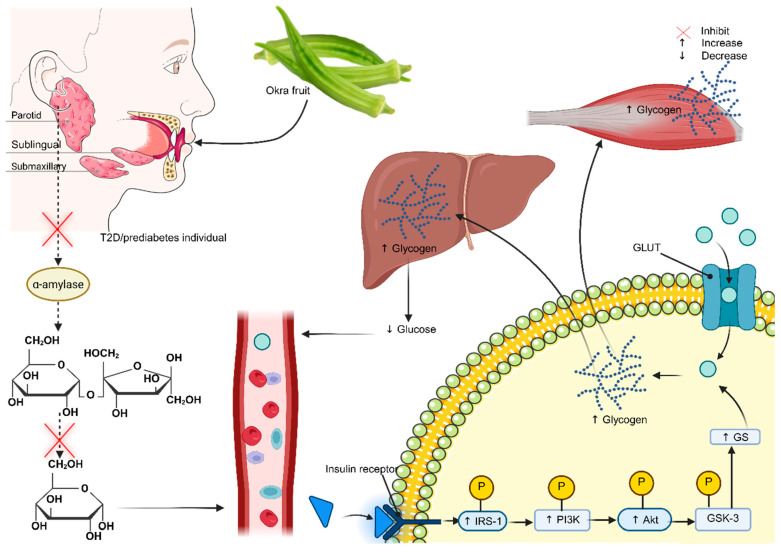
Potential mechanism by which okra lowers blood glucose in prediabetes and type 2 diabetes [61,62]. GS: glycogen synthase; IRS1: insulin resistance substrate 1; PI3K: phosphoinositide 3-kinase; Akt: protein kinase; GSK3: glycogen synthase kinase 3; P: phosphate; GLUT: glucose transporter; PPAR-α: peroxisome proliferator-activated receptor alpha. Diagram created using BioRender and Biocon.

In contrast, another study reported a 0.66-fold increase in HOMA-IR after okra treatment compared with baseline, suggesting that okra was ineffective in improving insulin sensitivity [37]. Among other factors, this study indicates that they did not control for participants’ dietary habits, physical activity, or medication adherence. Altogether, these factors may contribute to reduced treatment efficacy. Despite understanding the potential effects of okra on markers of glycemia and insulin resistance, the mechanism by which it regulates these processes remains an area yet to be fully explored, especially in clinical trials. However, evidence from in vitro studies suggests that okra modulates this function by altering the function of various carbohydrases. For instance, fresh okra showed stronger inhibition of α-amylase, with inhibition dose-dependent [54]. Additionally, the polyphenol-rich extract of *Abelmoschus esculentus* seed also proved to have more substantial α-amylase-inhibiting potential [55]. It is worth noting that blood glucose concentration may rise depending on how effectively α-amylase and β-glucosidase break down carbohydrates. Consistently, okra increased the inhibition rates of α-amylase and α-glucosidase by 28.6% and 31.6%, respectively [56]. This is further supported by other studies that confirm okra’s inhibitory effect on carbohydrases [57,58,59,60]. This activity reduces the influx of glucose into the bloodstream, thus reducing hyperglycemia (Figure 6).

Another mechanism reported by Liao et al. (2019) in the T2D model demonstrated that okra polysaccharides upregulate the phosphoinositide 3-kinase/protein kinase B (PI3K/Akt) pathway, further regulating glucose homeostasis and insulin signaling [63] (Figure 6). This activity supports the antihyperglycemic potential of okra, particularly in the management of diabetes. Additionally, evidence from murine studies has shown that okra maintains glucose homeostasis by activating the peroxisome proliferator-activated receptor (PPAR) pathway, which upregulates the expression of PPAR-α, β, and γ [64,65,66]. Notably, PPAR-α regulates gluconeogenesis in the liver, while PPAR-β enhances glucose utilization in various tissues, and PPAR-γ regulates glucose uptake in adipocytes. Together, these receptors enhance uptake and utilization, thereby improving insulin sensitivity [67].

**Figure 7:**
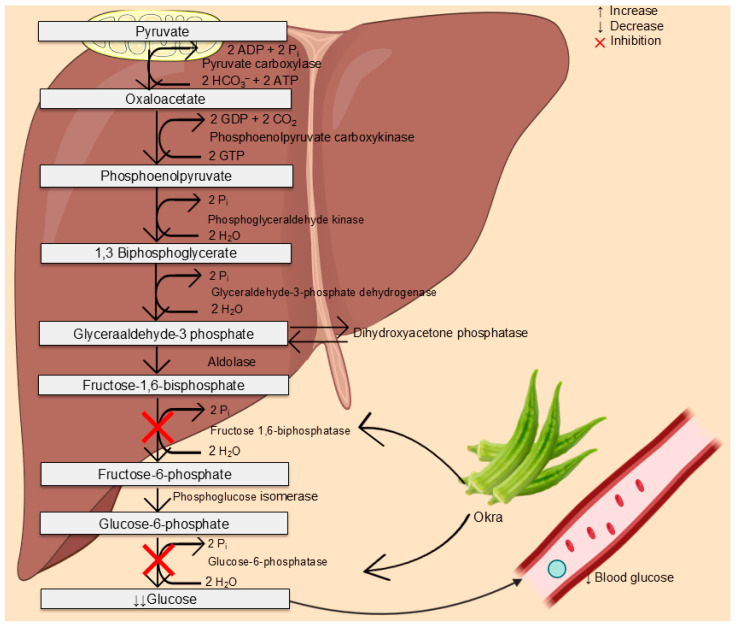
Potential mechanism by which okra lowers blood glucose in prediabetes and type 2 diabetes [61,62]. P_i_: phosphate ion; H_2_O: water; ADP: adenosine diphosphate; ATP: adenosine triphosphate; GTP: guanosine triphosphate. Diagram created using BioRender, Biocons, and Microsoft PowerPoint.

On the other hand, okra appears to exert its antidiabetic effect by inhibiting enzymes involved in gluconeogenesis, ultimately reducing glucose production from glycerol, pyruvate, lactate, and amino acids. This process is facilitated by the activation of pyruvate carboxylase, phosphoenolpyruvate carboxykinase, fructose-1,6-bisphosphatase, and glucose-6-phosphatase, all of which result in the formation of glucose [68]. Inhibition of fructose-1,6-bisphosphatase and glucose-6-phosphatase following supplementation with 200 and 400 mg/kg of okra, as observed in a rat model of hyperlipidemia, further supports its antihyperglycemic potency (Figure 7) [61,62].

Moreover, evidence from in vitro studies indicates a high phytochemical content, including alkaloids, quercetin, saponins, flavonoids, phenols, and anthraquinones [15,69,70]. Of these compounds, quercetin appears to lower glucose levels by increasing insulin secretion, thereby improving insulin resistance and enhancing glucose uptake in muscle through the GLUT4-mediated pathway, which involves PI3K and adenosine monophosphate-activated protein kinase (AMPK) [71]. This activity mediates hepatic glucose homeostasis by inhibiting enzymes involved in the gluconeogenesis pathway. This subsequently enhances hepatic glycogen synthesis and pancreatic islet regeneration, thereby promoting insulin secretion. Similarly, saponins improve insulin secretion by protecting β-cells and reducing insulin resistance by modulating the PI3K/Akt signaling pathway [72]. The high levels of phenols reportedly promote antihyperglycemic effects by inhibiting α-amylase and α-glucosidase, thereby activating the AMPK pathway, increasing glucose uptake, and enhancing insulin signaling [73]. An in vitro study has demonstrated the antidiabetic properties of anthraquinone, which upregulate IRS-1, PI3K, and Akt signaling pathways, commonly associated with the inhibition of α-glucosidase and the activation of PPAR-γ [74]. Altogether, these compounds may improve insulin resistance by activating the AMPK and PI3K/Akt pathways. Previous findings have also shown that the anti-hyperlipidemic and antioxidative properties of *Abelmoschus esculentus* ameliorate hyperglycemia by restoring insulin sensitivity, enhancing glucose uptake, reducing hepatic glucose output, and thereby protecting β-cells from damage [75,76].

While the current study demonstrates the potential benefits of okra in regulating glucose homeostasis, it is essential to acknowledge some potential limitations. For example, the study was registered with PROSPERO to ensure transparency and prevent duplication. The inclusion of 19 clinical studies, which comprise randomized controlled trials and quasi-experimental studies, introduces methodological variation in the evidence presented. However, through thorough subgroup analysis, we have accounted for this variation. The studies included used different doses and forms of okra (fruit, juice, soaked water, powdered capsules, or powder in meals), different processing methods for okra, and varying intervention durations. It is also important to highlight that the efficacy of a drug or any treatment depends on patient adherence. However, assessing adherence was challenging, as most included studies did not specify patients’ adherence to okra treatment. Furthermore, a significant concern is the distribution of publications by the location where the studies were conducted, as all studies were published in Asia. We also included one study published in a language other than English, and we translated its findings using an online translation service. While we included different conditions, it is important to note that only one study included prediabetics, while the rest included T2D. Hence, subgroup analysis on this factor was not possible. Although other studies mentioned the treatment that patients were on in addition to okra, others did not specify this, thus making it difficult to understand and draw a conclusion about herb-drug interference, which could interfere with the safety and efficacy of the treatment. Through the subgroup, we also found that publications from studies with moderate quality might have contributed to bias, especially on FBG.

## 5. Conclusions and Recommendations

The evidence presented revealed that okra supplementation in individuals with prediabetes and T2D may significantly improve glycemia by reducing FBG and HbA1c, and reduce insulin resistance by lowering HOMA-IR. However, no effect on insulin levels was observed. Overall, the results suggest that okra can be used as an adjunct therapy to control glycemia and insulin resistance in these patients. Therefore, future clinical trials should investigate the effect of okra on insulin resistance to provide a solid conclusion on its therapeutic efficacy in individuals with prediabetes and T2D. Given the small sample sizes in individual trials, we recommend that future studies recruit more patients, especially those with prediabetes, to mitigate complications and prevent the development of T2D. We also recommend that future trials should standardize the optimal dose of okra and duration of intervention that will be more effective in the management of hyperglycemia among those with prediabetes and T2D.

## Figures and Tables

**Table 1:** The geographical and demographic information of the included studies.

Authors, Reference	Country	Study Design	Condition and Sample	Intervention	Control	Age in Intervention	Age in Control	BMI in Intervention (kg/m^2^)	BMI in Control	Gender in Intervention (m/f)	Gender in Control (m/f)	OUTCOME Reported
Moradi et al. [42]	Iran	Double-blinded, single-center, randomized clinical trial	T2D	25 individuals, 10 g of okra powder (100 g of fresh okra blended in 150 g of yogurt) for 8 weeks	23 individuals on placebo (yogurt plus a consumable color)	54.26 ± 7.62	53.33 ± 7.35	24.90 ± 3.94	25.65 ± 3.46	9/21	7/23	FBG, HbA1c, insulin, HOMA-IR
Saatchi et al. [41]	Iran	Double- blinded randomized clinical trial.	T2D	50 individuals on okra (1000 mg of okra powder, microcrystalline cellulose, and magnesium stearate) every 6 h with food for 8 weeks	50 individuals on a placebo (microcrystalline cellulose and magnesium stearate)	57.7 ± 9.7	58.3 ± 9.2	30.2 ± 4.3	31.1 ± 4.1	23/26	13/37	FBG, HbA1c
Tavakolizadeh et al. [40]	Iran	Double-blind randomized, placebo-controlled, clinical trial	T2D	48 individuals on okra powder (2 capsules of 500 mg of powdered okra fruit three times) 30 min before each major meal with a glass of water for 12 weeks	46 individuals on placebo (microcrystalline cellulose, the same number of capsules per day containing 500 mg toasted flour) for 12 weeks	53.8 ± 3.7	52.8 ± 4.6	28.6 ± 2.05	29.5 ± 3.36	17/31	13/33	FBG, HbA1c, insulin, HOMA-IR
Nikpayam et al. [39]	Iran	Triple-blind placebo-controlled randomized clinical trial	T2D with DN	30 individuals on okra (one 125 mg capsule of containing 80 mg dried okra extract, 3.2 mg Avicel, and 0.8 mg magnesium stearate) for 10 weeks	25 individuals on placebo (one 125 mg capsule of carboxymethyl cellulose) for 10 weeks.	62 ± 7.0	61.6 ± 8.5	30.35 ± 5.05	28.64 ± 3.17	10/20	6/19	FBG, HbA1c, insulin, HOMA-IR
Hesamzadeh et al. [37]	Iran	Double-blind, placebo-controlled, multi-center, two- group clinical trial	T2D	27 individuals on okra (2500 mg capsule of dried okra fruit powder), three times daily for 60 days	26 individuals on a placebo (microcrystalline cellulose) for 60 days	53.41 ± 6.36	52.73 ± 8.69	29.96 ± 5.69	29.85 ± 5.05	8/19	4/22	FBG, HbA1c, insulin, HOMA-IR
Raiesifar et al. [36]	Iran	Double-blinded randomized clinical trial	T2D	30 individuals on diabetic nutrition program with 20 g of oral okra fiber for 28 days	30 individuals in the control diabetic nutrition program	18 and above	18 and above	NR	NR	22/8	16/14	FBG, HbA1c
Putriana et al. [50]	Indonesia	Single-blinded randomized controlled trial	T2D	14 individuals on okra pudding (200 g) for 6 weeks	14 individuals on a pudding for 6 weeks	19–59	19–59	23.43 ± 4.01	21.86 ± 3.23	1/13	3/11	FBG, HbA1c
Rad et al. [35]	Iran	Triple-blind, randomized, clinical trial	T2D	44 individuals on herbal remedy (200 mg capsule with *A. esculentus* fruit, aerial parts of *E. billardieri*, seed of *U. dioica*, *T. foenum-* *graecum*, bark of *C. zeylanicum*, and *R. canina* fruit, for 3 months	44 individuals on a placebo (microcrystalline cellulose after the main meal with their daily drugs such as Metformin and Sulfonylurea) for three months	59.02 ± 9.73	57.61 ± 11.55	29.54 ± 4.33	28.84 ± 4.16	13/31	16/28	FBG, HbA1c
Shivasankari et al. [38]	India	Quasi-experimental	T2D	30 individuals on okra (soaked medium-sized lady’s finger in 200 mL of water overnight) drink on an empty stomach in the morning	30 individuals on routine diabetic medications	40–60	40–60	NR	NR	19/11	19/11	FBG
Radhika et al. [34]	India	RCT	T2D	56 individuals on okra (40 mg of lady’s fingers soaked in 200 mL of warm water for 12 h, overnight) taken for 15 days	57 individuals on oral antidiabetic drug for 15 days	53.3 ± 8.7	55.5 ± 9.8	25.9 ± 3.5	25.6 ± 3.7	23/33	14/43	FBG
Sathiyabama et al. [43]	India	Quasi-experimental	T2D	35 individuals on okra (2 mg/day of lady’s finger juice before breakfast for 21 days	35	40–60	40–60	NR	NR	18/17	14/21	FBG
Shah et al. [48]	Pakistan	Quasi-experimental	T2D	27 individuals on okra (200 g of raw ladyfinger in divided doses) for three months	30 individuals on a placebo (capsules containing ground wheat shell only)	NR	NR	NR	NR	NR	NR	FBG
Gomathi et al. [49]	India	Randomized pilot clinical trial	T2D	5 individuals on okra and metformin (200 mL of okra juice in the morning one time on an empty stomach for 90 days	5 individuals on metformin	40–60	40–60	26.97 ± 1.06	25.66 ± 5.26	2/3	1/4	FBG, HbA1c
Khodija et al. [44]	Indonesia	Clinical trial study	T2D with hypercholesterolemia	12 individuals on boiled okra (40 g) for breakfast for 14 days	16	45–65	45–65	NR	NR	1/11	6/10	FBG
Khodija et al. [44]	Indonesia	Clinical trial study	T2D	12 individuals on steamed okra (40 g) for 14 days	16	45–65	45–65	NR	NR	1/11	6/10	FBG
Haryati et al. [45]	Indonesia	Quasi experimental	T2D	Three pieces of fresh okra fruit soaked in 250 mL water for 10 h. Taken daily before breakfast.	NR	NR	NR	NR	NR	4/11	4/11	FBG
Davis et al. [46]	India	Quasi experimental	T2D	20 individuals on lady’s fingers	20	45–60						FBG
Rahman et al. [47]	Bangladash	Cross-over trial	T2D	10 individuals on lady’s fingers for 180 min	10 individuals were given white bread	41.85 ± 5.9	41.85 ± 5.9	24.14 ± 2.45	24.14 ± 2.45	5/5	5/5	FBG
Septiningtiyas et al. [33]	Indonesia	Quasi experimental	T2D	18 individuals on the okra juice for 7 days	18 control group was not given any intervention, and after the study, they were given okra juice for 7 days.	66.06 ± 3.04	66.17 ± 4.32	NR	NR	5/13	5/13	FBG
Chen et al. [32]	China	RCT	Prediabetes	30 individuals on *Hibiscus esculentus* (20 g *of Hibiscus esculentus* dried fruit tea divided into three servings, brewed with 200 mL of warm water, to be consumed after breakfast, lunch, and dinner with chewed pulp) in addition to lifestyle changes for 60 days	30 individuals continued changing their lifestyle	40.8 ± 5.4	41.4 ± 5.9	NR	NR	18/12	14/16	FBG, HbA1c, insulin, HOMA-IR

T2D: type 2 diabetes; RCT: randomized controlled trial; BMI: body mass index; FBG: fasting blood glucose; HbA1c: glycated hemoglobin; NR: not reported; HOMA-IR: homeostatic model assessment of insulin resistance.

## Data Availability

All data supporting this manuscript are provided in the Supplementary Materials.

## Supplementary Materials

The following supporting information can be downloaded at: https://www.mdpi.com/article/10.3390/plants15050817/s1, Supplementary File S1: PRISMA checklist; Supplementary File S2 Figure S1: Risk of bias of included studies. A: ROB of randomized controlled trials; B: ROB of non-randomized studies; Table S1: Literature search, Table S2: Sensitivity analysis; Table S3: Subgroup analysis.
