# Supplementary material for: Investigating the Impact of Abelmoschus esculentus on Glycemia and Insulin Resistance in Type 2 Diabetes and Prediabetes
Source: Plants (Basel). 2026 Mar 7;15(5):817. doi: 10.3390/plants15050817 (PMC12986651; doi:10.3390/plants15050817)
Supplement: Supplementary file 1 [file plants-15-00817-s001.zip › Supplementary File S2.pdf]

Table S1: Literature search

Database	Exact search	Records identified
PubMed	((((okra[MeSH Terms]) OR (abelmoschus esculentus[MeSH Terms])) OR (hibiscus esculentus[MeSH Terms])) OR (ladies finger[MeSH Terms])) AND (diabetes[MeSH Terms]) Filters: Clinical Trial, Randomized Controlled Trial	6
Scopus	(TITLE-ABS-KEY (okra) OR TITLE-ABS-KEY (abelmoschus esculentus) OR TITLE-ABS-KEY (hibiscus esculentus) OR TITLE-ABS-KEY (ladies finger) AND TITLE-ABS-KEY (diabetes) AND TITLE-ABS-KEY (randomized controlled trial) AND TITLE-ABS-KEY (clinical study)) AND (LIMIT-TO (DOCTYPE, "ar"))	10
ScienceDirect	abelmoschus esculentus AND diabetes mellitus AND randomized controlled trials	12
Web of Science	okra OR abelmoschus esculentus AND diabetes AND randomized controlled trial	17

Table S2: Sensitivity analysis

**HOMA-IR**			
**Study**	**MD**	**95%CI**	I^2
Moradi et al	–0.46#	–1.02 to 0.10	25.1
Chen et al.	–0.55#	–1.18 to 0.07	39.9
Tavakolizadeh et al.	–0.56*	–1.06 to –0.05	34
Nikpayam et al.	–0.74**	–1.24 to –0.24	0
Hesamzadeh et al.	–0.71*	–1.22 to – 0.19	27.5
**Insulin**			
**Study**	**SMD**	**95%CI**	I^2
Moradi et al.	0.14#	–0.10 to 0.39	6.1
Chen et al.	–0.02#	–0.49 to 0.23	0
Tavakolizadeh et al.	0.14#	–0.12 to 0.41	34.3
Nikpayam et al.	0.07#	–0.18 to 0.31	46.1
Hesamzadeh et al.	0.02#	–0.23 to 0.26	35.5
**HbA1c**			
**Study**	**MD**	**95%CI**	I^2
Gomathi et al.	–0.55*	–1.12 to –0.01	92.9
Moradi et al.	–0.86*	–1.49 to –0.23	94.4
Saatchi et al.	–0.80*	–1.46 to –0.13.	94.7
Chen et al.	–0.85*	–1.48 to –0.23	94.6
Tavakolizadeh et al.	–0.77*	–1.41 to –0.13	94.8
Nikpayam et al.	–0.81*	–1.43 to –0.18	94.7
Hesamzadeh et al.	–0.89*	–1.51 to –0.27	94.3
Raiesifar et al.	–0.84*	–1.50 to –0.18	92.4
Rad et al.	–0.64#	–1.18 to 0.10	88.8
Putriana et al	–0.71*	–1.32 to –0.10	94.7
**FBG**			
**Study**	**SMD**	**95%CI**	I^2
Rahman et al.	–0.73***	–1.09 to –0.37	86.6
Davis et al.	–0.72***	–1.07 to –0.38	87.1
Haryati et al.	–0.70***	–1.04 to –0.35	87.2
Khodija (A) et al.	–0.64***	–0.97 to –0.31	86.4
Khodija (B) et al.	–0.64***	–0.97 to –0.31	86.4
Gomathi et al.	–0.67***	–1.00 to –0.33	87.0
Moradi et al.	–0.71***	–1.06 to –0.36	87.2
Sathiyabama et al.	–0.67***	–1.02 to –0.33	86.8
Radhika et al.	–0.67***	–1.02 to – 0.32	86.6
Shah et al.	–0.69***	–1.04 to – 0.34	87.1
Saatchi et al.	–0.70***	–1.05 to – 0.34	87.2
Chen et al.	–0.75***	–1.09 to – 0.42	85.8
Tavakolizadeh et al.	–0.67***	–1.02 to – 0.32	86.6
Nikpayam et al.	–0.71***	–1.06 to – 0.36	87.3
Shivasankari et al.	–0.71***	–1.06 to – 0.36	87.2
Hesamzadeh et al.	–0.77***	–1.09 to – 0.44	85.2
Raiesifar et al.	–0.78***	–1.09 to – 0.47	83.7
Rad et al.	–0.61***	–0.92 to – 0.30	83.4
Septiningtiyas et al.	–0.70***	–1.05 to – 0.35	87.3
Putriana et al.	–0.72***	–1.07 to –0.38	87.1

*** $p < 0.0001$, ** $p < 0.01$, * $p < 0.05$, # $p > 0.05$.

Table S3: Subgroup analysis

Outcome	Subgroups	Class	Studies	Effect size	I^2
FBG	Condition	T2D	19	–0.75 (–1.09 to –0.42)	85.8
		Prediabetes	1	0.34 (–0.17 to 0.85)	N/A
HbA1c	Condition	T2D	9	–0.85(–1.48 to –0.23)	94.6
		Prediabetes	1	0.00 (–0.83 to 0.83)	N/A
FBG	Country	Bangladesh	1	–0.34 (–0.17 to 0.85)	NA
		China	1	–0.27 (–0.55 to 0.01)	N/A
		India	5	–0.85 (–1.28 to –0.41)	63.1
		Iran	7	–0.53 (–1.25 to –0.19)	93
		Indonesia	5	–1.08 (–1.79 to –0.38)	74.5
		Pakistan	1	–0.92 (–1.47 to –0.37)	N/A
HbA1c	Country	China	1	–0.00 (–0.83 to 0.83)	NA
		India	1	–2.62 (–3.18 to –2.06)	N/A
		Iran	7	–0.54 (–1.16 to 0.09)	94.5
		Indonesia	1	–1.18 (–3.65 to 0.03)	N/A
FBG	Quality	High	7	–0.53 (–1.25 to –0.19)	93.0
		Low	10	–0.73 (–1.16 to –0.30)	81.2
		Moderate	3	–0.98 (–1.32 to – 0.65)	0
HbA1c	Quality	High quality	7	–0.54 (–1.16 to 0.09)	94.5
		Low quality	3	–1.47. (–3.41 to 0.47)	92.4

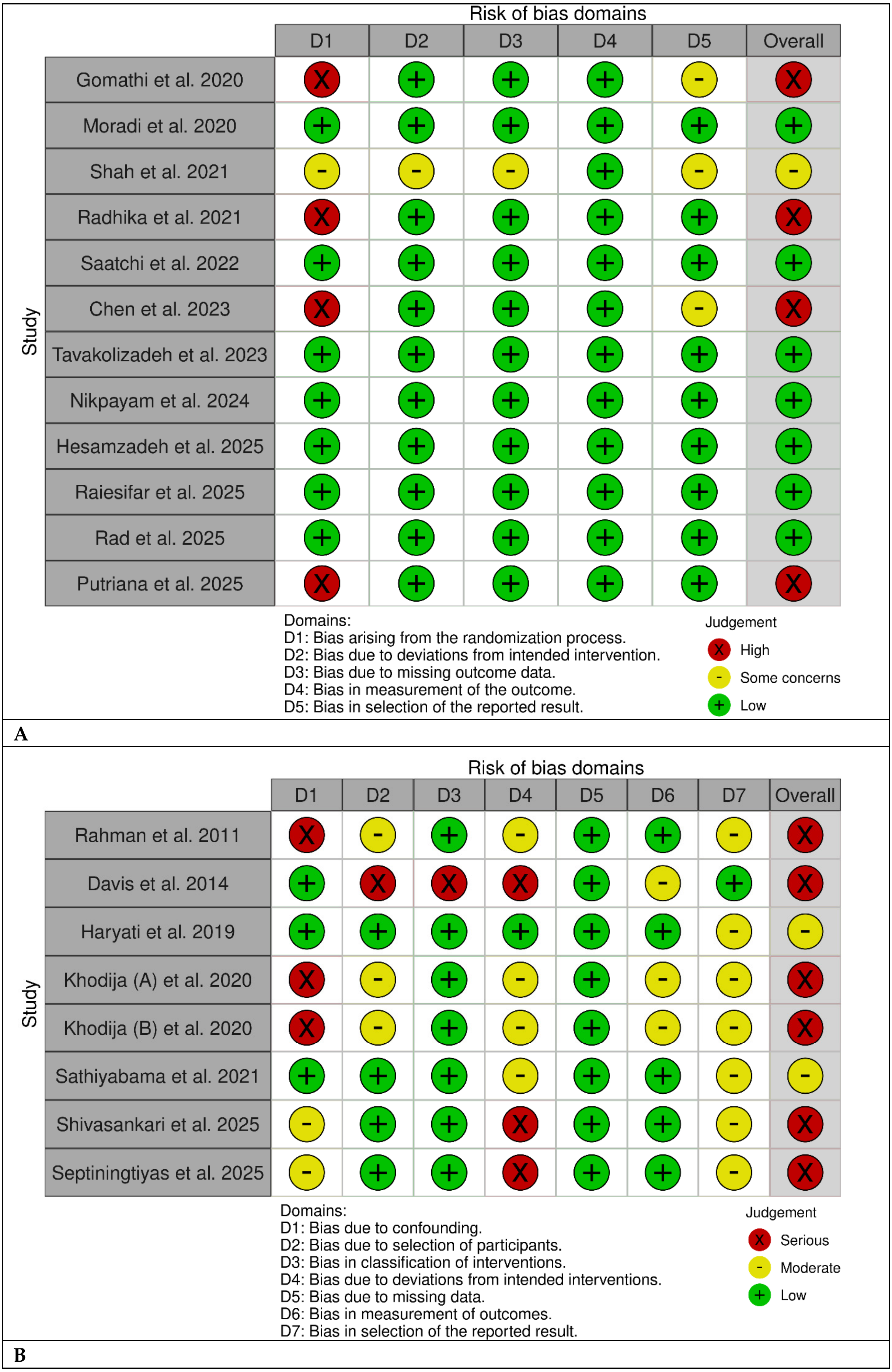

Figure S1: Risk of bias of included studies. A: ROB of randomized controlled trials [32, 34–37, 39–42, 48–50]; B: ROB of non-randomized studies [33,38,43–47].
